# Real-world experience with the pentaspline pulsed field ablation system: one-year outcomes of the FARADISE registry

**DOI:** 10.1093/europace/euaf182

**Published:** 2025-09-01

**Authors:** Lucas V A Boersma, Gábor Széplaki, Antonio Dello Russo, Ignacio García-Bolao, Michael Efremidis, Nándor Szegedi, Stephan Willems, Haris Haqqani, Estelle Gandjbakhch, Francesco Solimene, George Andrikopoulos, Martin Fiala, Pascal Defaye, Armin Luik, Patrick Lugenbiel, Lars Eckardt, Alexandre Ouss, Jean-Manuel Herzet, Javier Ramos Maqueda, Sélim Abbey, Joaquín Osca, Azlan Hussin, Nele Cielen, Madeline Johnson, Elizabeth M Albrecht, Brad S Sutton, Johan Vijgen

**Affiliations:** Cardiology Department, St. Antonius Hospital, PO 2500, 3430 EM, Nieuwegein, The Netherlands; Mater Private Hospital and Royal College of Surgeons in Ireland, Dublin, Ireland; Biomedical Science and Public Health Department, Polytechnic University of Marche, AOUM Hospital, Ancona, Italy; University Clinic of Navarra, Navarra, Spain; Onassis Cardiac Surgery Center, Kallithea, Greece; Heart and Vascular Center, Semmelweis University, Budapest, Hungary; Asklepios Clinic St. Georg, Hamburg, Germany; Prince Charles Hospital, Chermside, Queensland, Australia; Sorbonne Université, APHP, Hospital La Pitié Salpetrière, Paris, France; Casa di Cura Montevergine SPA, Mercogliano, Italy; Henry Dunant Hospital Center, Athens, Greece; Neuron Medical, Brno, Czech Republic; CHU Grenoble—Hopital Michallon, Grenoble, France; Staedtisches Klinikum Karlsruhe, Karlsruhe, Germany; Department of Cardiology, Medical University Hospital Heidelberg, Heidelberg, Germany; Department of Cardiology—Electrophysiology, University Hospital of Münster, Münster, Germany; Catharina Ziekenhuis, Eindhoven, The Netherlands; Centre Hospitalier Régional de la Citadelle, Liège, Belgium; Hospital Clinico Universitario Lozano Blesa, Zaragoza, Spain; Hopital Prive du Confluent, Nantes, France; Hospital Universitario y Politécnico La Fe, Valencia, Spain; Institut Jantung Negara, Kuala Lumpur, Malaysia; Boston Scientific, St. Paul, MN, USA; Boston Scientific, St. Paul, MN, USA; Boston Scientific, St. Paul, MN, USA; Boston Scientific, St. Paul, MN, USA; Jessa Hospital, Hasselt, Belgium

**Keywords:** Pulsed field ablation, Atrial fibrillation, Real-world, Learning curve

## Abstract

**Aims:**

Clinical studies with protocol-mandated workflow and monitoring have analysed performance of pulsed field ablation (PFA) for treating atrial fibrillation (AF). The FARADISE registry captures global use of the pentaspline PFA catheter in real-world clinical practice with a follow-up of 3 years.

**Methods and results:**

FARADISE is a prospective, non-randomized, multi-national registry (NCT05501873) that enrolled subjects clinically indicated for ablation using the pentaspline PFA catheter per medical judgement and hospital standard-of-care. Procedural characteristics, safety, and clinical effectiveness up to 12-months were collected. In total, 1158 AF patients received PFA across 48 centres in 21 countries (64 ± 11 years, 33% female, 90% *de novo*, 65% paroxysmal AF). Pulmonary vein isolation (PVI)-only procedures were performed in 80.8% of paroxysmal vs. 57.5% for non-paroxysmal patients (*P* < 0.01). Median procedure, left atrial dwell, and fluoroscopy times were 51[40–70], 31[24–41], and 12[8–17] min, respectively. The rate of early onset serious adverse events was 1.5% and did not differ by ablation strategy or AF indication. At 1-year, clinical effectiveness was 80.8% for paroxysmal AF and 67.7% for non-paroxysmal AF, with no difference within indication by lesion set (paroxysmal: 81.2% PVI-only vs. 79.0% PVI+, *P* = 0.65; non-paroxysmal: 67.5% PVI-only vs. 67.7% PVI+, *P* = 0.79). Acute results reinforce a short procedural learning curve with no difference in 1-year effectiveness by operator experience.

**Conclusion:**

The FARADISE registry provides a snapshot of real-world clinical use of the pentaspline PFA catheter. Acute results demonstrate favourable procedural and safety outcomes regardless of AF indication. One-year outcomes are encouraging, with no differences seen within indication based on ablation strategy.

## Graphical Abstract

**Graphical Abstract:**
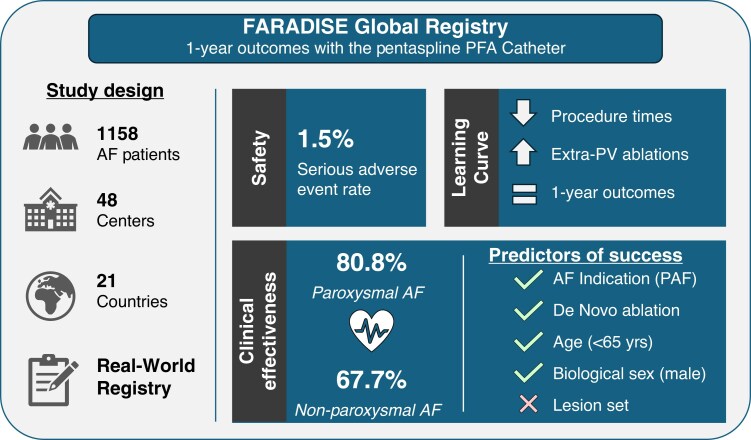

What’s new?

- This is the first global, prospective, real-world registry to report 1-year outcomes using the pentaspline pulsed field ablation (PFA) system across 48 centres in 21 countries.
- The study demonstrates high clinical effectiveness at 12-months: 80.8% in paroxysmal and 67.7% in non-paroxysmal atrial fibrillation (AF) patients, with no difference observed in outcomes based on ablation strategy.
- A low rate of serious adverse events (1.5%) was observed, with no deaths, atrio-oesophageal fistula, or pulmonary vein stenosis.
- Procedural efficiency improved with operator experience, yet 1-year effectiveness remained consistent across all experience levels, highlighting a short learning curve with the pentaspline PFA catheter.

## Introduction

Atrial fibrillation (AF) continues to be the most prevalent cardiac arrhythmia globally^1,2^ with an increasing number of patients opting for AF ablation procedures. The evidence surrounding rhythm control as an effective strategy for maintaining sinus rhythm and preventing AF progression is growing^3–6^ and recent guidelines have endorsed AF ablation as a first line treatment for select patients.^2^

Pulsed field ablation (PFA) has quickly gained traction since its commercial introduction into AF ablation in 2021. Single centre registries and retrospective evaluation during early use of this technology have been promising.^7–10^ The demonstration of a favourable safety profile has been one of the primary drivers for the clinical adoption of PFA in Europe.^9,11^ Further, several studies have shown clinical efficiency and short learning curve despite most operators being dedicated radiofrequency or cryoballoon users and years of training with thermal ablation modalities, making PFA a seemingly predictable choice amongst operators.^8,9^

Global commercial availability of this technology provides a growing understanding of wide-scale adoption across geographies. However, detailed acute, mid-term, and long-term prospective data on real-world use of the pentaspline PFA catheter is still limited (especially across the wide range of geographies not among the early adopters). The FARADISE global registry (NCT05501873) seeks to capture the real-world use of the pentaspline PFA catheter and characterize long-term clinical outcomes. As FARADISE enrolment has closed, we now report on the procedural data and clinical outcomes at 12-month follow-up.

## Methods

FARADISE is a prospective, non-randomized, single-arm registry enrolling subjects clinically indicated for an AF ablation procedure using the pentaspline PFA catheter. This study was performed in accordance with the European Medical Device Regulation, ISO 14155 Clinical Investigation of Medical Devices or Human Subjects—Good Clinical Practice, and ethical principles consistent with the Declaration of Helsinki. Ethics Committee approval was obtained at all investigational sites. Informed written consent was obtained from all trial participants before enrolment.

The goal of this study was to enrol 1000–1500 patients from up to 100 sites, with a maximum of 40 patients enrolled at each site. Sites were included from Europe, Middle East, and Asia Pacific where the FARAPULSE PFA system is commercially available. Operators with varying levels of experience using the pentaspline catheter participated in this study.

### Patient population

Eligible subjects included those who were clinically indicated for AF ablation with FARAPULSE PFA system per physician’s medical judgement and per hospital’s standard-of-care. Exclusion criteria included subjects with recent myocardial infarction or cerebral vascular accident, known or suspected atrial myxoma, current inter-atrial baffle or patch, active systemic infection, any prosthetic heart valve, ring, or repair, unable to tolerate anti-coagulation therapy, presence of known atrial thrombus, inability to obtain vascular access, women who were pregnant or planned to become pregnant, AF secondary to electrolyte imbalance, thyroid disease, alcohol, or other reversible non-cardiac causes. Subjects with a contraindication to an invasive electrophysiology procedure or with a life expectancy of 1 year or less were excluded. Subjects were not allowed to be enrolled in another investigational study or registry that would interfere with the current study.

### Pulsed field ablation procedure

Ablation procedures were carried out per each hospital’s standard-of-care. Procedures were performed either under general anaesthesia or sedation. Use of adjunctive technologies such as 3D electroanatomical mapping and intra-cardiac echocardiography were used at the operator’s discretion. The pentaspline PFA catheter (Farawave™, Boston Scientific, Marlborough, MA) was introduced into the left atrium (LA) via a steerable sheath (Faradrive™). Pulsed field applications were delivered using the PFA generator (Farastar™) with a voltage output of 1.8–2.0 kV. Each application consisted of a biphasic waveform with five consecutive pulse trains delivered over 2.5 s. Following PFA of the PVs, confirmation of isolation was performed with entrance block. Additional lesions outside of the PVs were performed based on hospital’s standard-of-care and per operator discretion. At the time of enrolment, the use of the Farapulse PFA System for the treatment of non-paroxysmal AF and for extra-pulmonary vein isolation (PVI) ablation was outside of the labelled indication.

### Endpoints

The primary safety endpoint is a composite of the device- and procedure-related serious adverse events (SAEs) out to day 7 and late onset SAEs out to 12-month follow-up. The early onset SAEs include death, myocardial infarction, persistent phrenic nerve palsy (PNP), stroke, transient ischaemic attack (TIA), peripheral or systemic thromboembolism, cardiac tamponade or perforation, pericarditis, pulmonary oedema, vascular access complications, heart block, and gastric motility/pyloric spasm disorder. The late onset SAEs include pulmonary vein stenosis and atrio-oesophageal fistula.

The primary efficacy endpoint is a composite of acute procedural success and chronic treatment success. The acute procedural success is defined as isolation of all clinically relevant PVs using the pentaspline PFA catheter and demonstrated isolation through at least PV entrance block testing. Chronic treatment success includes freedom from documented AF or new onset atrial flutter (AFL) or atrial tachycardia (AT) events >30 s with any approved clinical arrhythmia monitoring system, or >10 s for 12-lead ECG, and freedom from interventions for AF or new onset AFL or AT, which include cardioversion, repeat ablation, AV node ablation, continued use of amiodarone, and change in anti-arrhythmic drugs (AADs; higher dose than any AAD used at baseline or any new AAD not documented at baseline), after a blanking period of 90 days post-index procedure. Note during follow-up, retaining post-ablation AADs is allowed per the investigator’s discretion during the blanking period.

### Additional endpoints

Given this registry captured real-world use of the pentaspline PFA catheter, the use of medication to manage patients is driven by disease and symptom management, comorbidities, and physician standard-of-care. Medication management of AADs and amiodarone were per standard-of-care and not protocol mandated. Additional analyses include freedom from atrial arrhythmia recurrence at 12 months (on and off anti-arrhythmic drugs), procedural times, rates of PFA-related adverse events, and assessment of operator variability and learning curve in different outcome measures.

### Follow-up

Following AF ablation, patients have follow-up visits at pre-discharge, 3, 6, 12, 24, and 36 months. Arrhythmia recurrence will be monitored using each hospital’s standard-of-care with the recommendation that patients receive 24-h Holter monitoring at 3 months and again at 1-, 2-, and 3-year follow-up. Here, we report on the data out to 12-month follow-up for all patients. Long-term follow-up is ongoing.

### Statistical analysis

Categorical variables are reported as absolute and relative frequencies and were compared using Fisher’s exact test. Continuous variables are reported as mean ± standard deviation in case of normal distribution and as median and inter-quartile range (first-quartile and third-quartile) otherwise. The continuous variables were compared using the non-paired Student’s *t*-test when normally distributed and the corresponding non-parametric test (Mann–Whitney *U* test) otherwise. Twelve-month recurrence was calculated using Kaplan–Meier methodology and compared using a log-rank test, and in cases where the proportional hazard assumption was violated compared with a Fleming–Harrington (1,1) test. For the clinical effectiveness through 12 months, a multi-variable analysis was performed with the demographics, along with the lesion set of PVI vs. PVI+, as potential covariates. Variables significantly associated with the outcome in a uni-variable Cox proportional hazards model at the 0.15 significance level were included in a multi-variable Cox proportional hazards model. Backwards selection with a 0.15 alpha exit criterion was used to determine the final multi-variable model for clinical effectiveness through 12 months. All statistical analyses were performed using SAS version 9.4 (SAS Institute), with a two-tailed *P*-value of <0.05 considered significant.

## Results

Patients clinically indicated for an AF ablation were enrolled in this registry for 1 year (from 24 March 2023 to 28 March 2024), and follow-up is currently ongoing with patients followed up to 3 years post-PFA ablation. Here, we report on the procedural data and clinical outcomes out to 12 months.

### Patients

**Figure 1:**
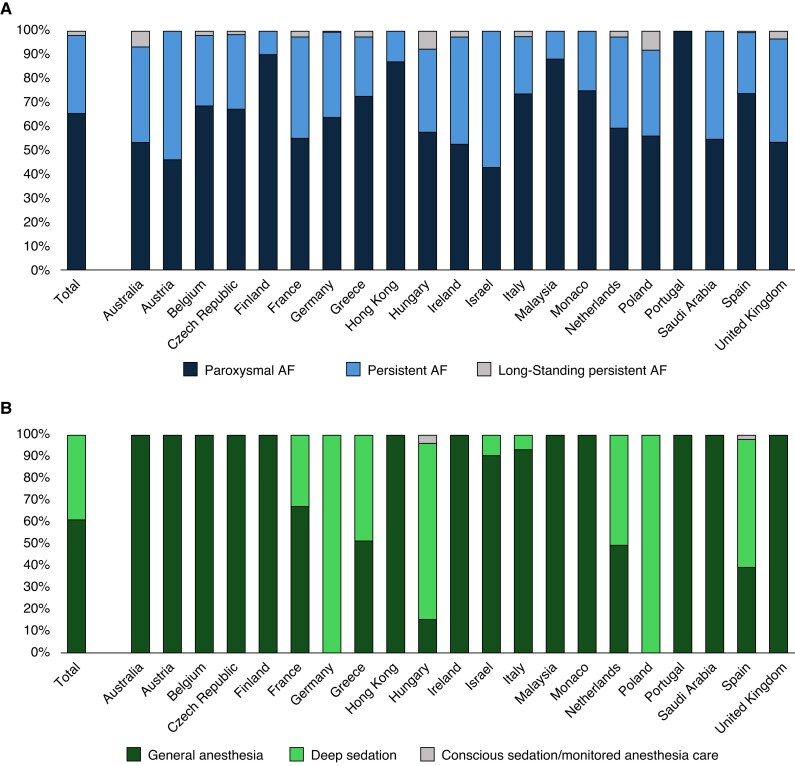
(*A*) Enrolment and atrial fibrillation indication by country. (*B*) Sedation technique by country.

In total, 1173 patients were enrolled across 48 centres in 21 countries (Europe, Asia, Australia, and Middle East). After enrolment, six subjects were found to be consent ineligible and six subjects did not have the study device inserted. There was one attempt subject who had the PFA device inserted, but no PFA applications were delivered. Ablation was performed using an RF catheter, and no adverse events or device deficiencies were noted for this subject. A total of 1160 patients received PFA treatment with the FARAPULSE system. Of these patients, two subjects did not have a history of AF and were not included in this analysis. Baseline characteristics of the 1158 AF patients treated with PFA in the real-world registry are shown in *Table 1*. Approximately 65.4% of the patients included in this registry had paroxysmal AF and 34.6% had non-paroxysmal AF (379 persistent AF and 22 long-standing (LS) persistent AF). The enrolment and AF indication by country is shown in *Figure 1A*. Patients averaged 64 years of age with 33.1% of the patients being female. The non-paroxysmal AF patients were older with larger atrial dimensions, lower ejection fractions, and higher rates of comorbidities.

**Table 1:** Patient demographics

	ALL	Paroxysmal AF	Non-paroxysmal AF	*P*-value
*N*	1158	757	401	
Age at enrolment, years	64 ± 11	63 ± 11	65 ± 10	<0.001
Sex (female), *N* (%)	383 (33.1)	276 (36.5)	107 (26.7)	<0.001
BMI (kg/m^2^)	28 ± 5	27 ± 5	29 ± 5	<0.001
LA diameter (cm)	4.2 ± 0.8 (*n* = 433)	4.0 ± 0.7 (*n* = 276)	4.5 ± 0.8 (*n* = 157)	<0.001
LVEF, %	60 (55, 64) (*n* = 873)	60 (55, 65) (*n* = 565)	55 (50, 60) (*n* = 308)	<0.001
CHADS-VASc	1.9 ± 1.4	1.7 ± 1.3	2.1 ± 1.4	<0.001
Comorbidities, *N* (%)				
Coronary artery disease	131 (11.3)	74 (9.8)	57 (14.3)	0.03
Diabetes	132 (11.4)	75 (9.9)	57 (14.2)	0.03
Heart failure	62 (5.4)	28 (3.7)	34 (8.5)	<0.001
Hypertension	611 (52.8)	378 (50.0)	233 (58.1)	0.009
Stroke/TIA	37 (3.2)	18 (2.4)	19 (4.7)	0.04
Sleep disordered breathing	104 (9.2)	60 (8.1)	44 (11.1)	0.11

Mean ± SD, Median (Q1, Q3), or *N* (%).

### Procedural characteristics

**Figure 2:**
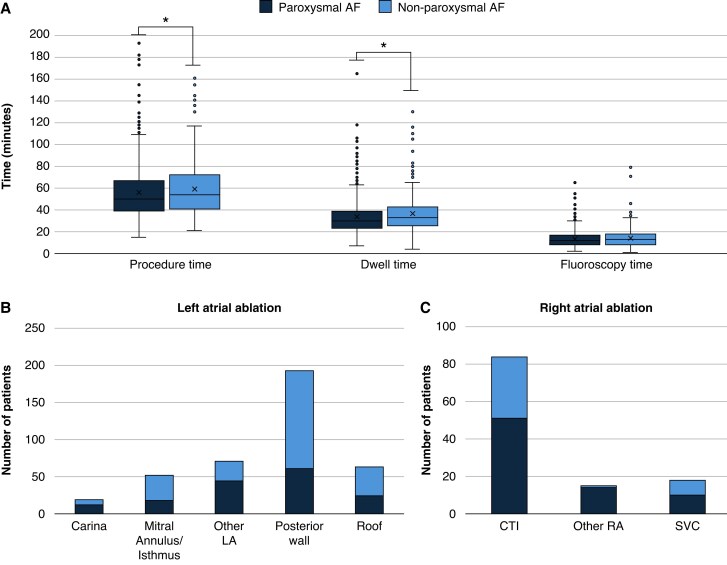
(*A*) Procedure times by atrial fibrillation indication. Non-pulmonary vein (*B*) left and (*C*) right atrial ablations performed by atrial fibrillation indication. * Indicates *P* < 0.01.

Procedural characteristics for the full patient population and the paroxysmal vs. non-paroxysmal groups are provided in *Table 2*. Most PFA procedures were performed under general anaesthesia (61.1%) or deep sedation (38.6%). As shown in *Figure 1B*, there is variation in sedation technique used for AF ablation based on geography due to national and institutional requirements, resources, and operator preference. Median skin-to-skin procedure times were 51 min [IQR: 40–70] with a median LA dwell time of 31 min [IQR: 24–41], and a median fluoroscopy time of 12 min [IQR: 8–17]. The procedure and LA dwell times were significantly higher in patients with non-paroxysmal AF compared to paroxysmal AF (*P* < 0.01; *Figure 2*). The majority of procedures were *de novo* ablations (90.1%), and 72.5% of all procedures had a PVI-only ablation strategy (80.8% of paroxysmal AF procedures vs. 57.5% of non-paroxysmal AF procedures, *P* < 0.01). The most common ablation targets outside of the PVs, performed with PFA or thermal ablation, were the posterior wall (PW; 16.5%), cavotricuspid isthmus (7.2%), left atrial roof (5.4%), and mitral isthmus (4.4%; *Figure 2*). Overall, 3D electroanatomical mapping and intra-cardiac echocardiography were only used in 14.5% and 16.1% of the procedures, respectively. Note that at the time of enrolment and procedures, the pentaspline PFA system did not have an integrated mapping system.

**Table 2:** Procedural characteristics

	ALL	Paroxysmal AF	Non-paroxysmal AF	*P*-value
Sedation, *N* (%)				
General anaesthesia	707 (61.1)	479 (63.3)	228 (56.9)	0.03
Deep sedation	447 (38.6)	274 (36.2)	173 (43.1)	
Conscious sedation/monitored anaesthesia care	4 (0.3)	4 (0.5)	0 (0.0)	
3D mapping, *N* (%)	168 (14.5)	95 (12.5)	73 (18.2)	0.01
ICE, *N* (%)	186 (16.1)	127 (16.8)	59 (14.7)	0.40
Procedure times (min)				
Skin-to skin procedure time	51 (40, 70)	50 (40, 67)	54 (41, 72)	0.01
Left atrial dwell time	31 (24, 41)	30 (23, 39)	33 (26, 43)	0.001
Fluoroscopy time	12 (8, 17)	12 (8, 17)	13 (8, 18)	0.13
*De novo* ablation, *N* (%)	1043 (90.1)	694 (91.7)	349 (87.0)	0.01
Lesion set, *N* (%)^a^				
PVI-only	840 (72.5)	610 (80.8)	230 (57.5)	<0.001
Extra-PV ablation	315 (27.2)	145 (19.2)	170 (42.5)	

Mean ± SD, Median (Q1, Q3), or *N* (%).

^a^Three subjects did not have PVI ablation performed at the index PFA procedure.

### Safety

Early onset procedure- and device-related SAEs, as defined per protocol, occurred in 17 patients (1.5%). SAEs are summarized in *Table 3*, which includes one stroke and one TIA occurring during the index procedure. For the subject who experienced a stroke, a CT scan was performed following the procedure, which showed no inter-cranial haemorrhage or evidence of infarction. The symptoms resolved and the patient was discharged from the hospital three days later. In the patient with the reported TIA, symptoms began immediately following the index procedure and lasted <24 h. A CT scan was performed with no signs of cerebral infarct. The patient’s hospital stay was prolonged with symptoms resolved at discharge. There was one phrenic nerve injury reported at the 1-month follow-up. For this patient, there was no PNP detected at the end of the index procedure. However, pneumonia was reported on the day of the index procedure. The patient had a fever with elevated white cell count but no localized symptoms. Chest X-ray showed signs of pneumonia in the right lobe. The patient was given an antibiotic and white cell counts normalized. The reported phrenic nerve injury was not classified as a per-protocol SAE as the onset date of the event was after the endpoint-specified 7-day window. Follow-up at 12 months showed partial recovery. Long-term follow-up is still ongoing, and the status of the phrenic nerve injury will be monitored. The most observed adverse event was significant vascular access complication (0.8%). There were no significant differences in the rate of SAEs in paroxysmal vs. non-paroxysmal AF patients (1.3% vs. 1.7%, respectively; *Table 3*) and no significant difference when split by lesion set into PVI-only (1.3%) vs. those receiving additional, non-PV ablations (1.9%). By 12-month follow-up, there were no reported deaths or instances of atrio-oesophageal fistula, PV stenosis, or oesophageal lesions. There was one reported instance of coronary spasm without clinical sequelae, confirmed using angiography, in a patient receiving PVI and CTI ablation. At 3-month follow-up, the patient returned for a coronary angiogram where no coronary abnormalities were observed. There were four reports of laboratory-confirmed haemolysis, only one of which was adjudicated as serious and required intervention (IV fluids). There was no requirement of haemodialysis as a result of haemolysis. Instances of haemolysis occurred in patients receiving 66, 93, 140, and 161 PFA applications. Additional information for each of the four haemolysis cases is provided in Supplementary material online, *Table S2*.

**Table 3:** Device- or procedure-related safety events

	All (*N* = 1158)	Paroxysmal AF (*N* = 757)	Non-paroxysmal AF (*N* = 401)	*P*-value
Per-protocol early onset SAEs, *N* (%)	17 (1.5)	10 (1.3)	7 (1.7)	0.61
Death	0 (0.0)	0 (0.0)	0 (0.0)	N/A
Myocardial infarction	0 (0.0)	0 (0.0)	0 (0.0)	N/A
Phrenic nerve injury (persistent)	0 (0.0)^a^	0 (0.0)^a^	0 (0.0)	N/A
Stroke	1 (0.1)	0 (0.0)	1 (0.2)	0.35
Transient ischaemic attack	1 (0.1)	1 (0.1)	0 (0.0)	1.00
Peripheral or systemic thromboembolism	0 (0.0)	0 (0.0)	0 (0.0)	N/A
Cardiac tamponade/perforation	4 (0.3)	2 (0.3)	2 (0.5)	0.61
Pericarditis	2 (0.2)	1 (0.1)	1 (0.2)	1.00
Pulmonary oedema	1 (0.1)	0 (0.0)	1 (0.2)	0.35
Heart block	0 (0.0)	0 (0.0)	0 (0.0)	N/A
Gastric motility/pyloric spasm disorder	0 (0.0)	0 (0.0)	0 (0.0)	N/A
Vascular access complication	9 (0.8)	6 (0.8)	3 (0.7)	1.00
Pulmonary vein stenosis	0 (0.0)	0 (0.0)	0 (0.0)	N/A
Atrio-oesophageal fistula	0 (0.0)	0 (0.0)	0 (0.0)	N/A
Additional relevant events, *N* (%)				
Coronary spasm	1 (0.1)	1 (0.1)	0 (0.0)	1.00
Haemolysis	4 (0.3)	1 (0.1)	3 (0.7)	0.12

^a^One persistent phrenic nerve injury was previously reported after the acute 7-day endpoint with partial recovery by 12 months. Follow-up is ongoing.

### One-year clinical effectiveness outcomes

At the time of this 12-month data snapshot (30 June 2025), 1128/1158 (97.4%) patients completed the 12-month follow-up visit. Overall, four patients have died during follow-up (not procedure- or device-related) and 26 patients missed their 12-month visit. The individual components of the composite efficacy endpoint are shown in *Table 4*. There were 16 patients (1.4%) who met the criteria for acute failure due to entrance block testing not being performed in one or more treated PVs. Of the 16 patients with acute failure, only three met a chronic failure component during follow-up (one for amiodarone use and two for AF recurrence). Overall, 244 patients had documented arrhythmia recurrences: 16.2% in the paroxysmal AF cohort and 30.2% in the non-paroxysmal AF cohort (*P* < 0.01). Repeat procedures and cardioversions were more often performed in non-paroxysmal AF patients compared to paroxysmal AF patients (9.0% vs. 4.9% and 13.0% vs. 6.3%, respectively). In total, there were 140 (12.1%) and 133 (11.5%) patients with documented AAD or amiodarone use, respectively. With this strict, off-AAD, single procedure efficacy definition, the 12-month treatment success was 64.1%. The rigorous treatment failure endpoint components, incorporating medication use and the 30-second arrhythmia recurrence definition, account for most of the composite endpoints met in this study. Given, this is a real-world registry, AAD use, including amiodarone, was not strictly monitored and left up physicians to manage based on standard-of-care.

**Table 4:** Primary efficacy endpoint components

	All *N* (%)	Paroxysmal AF *N* (%)	Non-paroxysmal AF *N* (%)	*P*-value
Endpoint components^a^				
Acute failure	16 (1.4)	13 (1.7)	3 (1.0)	0.29
AF/AFL/AT recurrence	244 (21.1)	123 (16.2)	121 (30.2)	<0.001
Repeat procedure	73 (6.3)	37 (4.9)	36 (9.0)	0.008
Cardioversion	100 (8.6)	48 (6.3)	52 (13.0)	<0.001
Amiodarone use	133 (11.5)	65 (8.6)	68 (17.0)	<0.001
AAD (escalation or new)	140 (12.1)	90 (11.9)	50 (12.5)	0.78
1-Year effectiveness				
Treatment success (off AADs)	742 (64.1)	524 (69.2)	218 (54.4)	<0.001
Treatment success (allowing AADs)^b^	886 (76.5)	613 (81.0)	273 (68.1)	<0.001

^a^Subjects can have more than one effectiveness failure component.

^b^Clinical effectiveness defined as freedom from acute failure, documented AF/AFL/AT recurrence, repeat procedure, and cardioversion.

**Figure 3:**
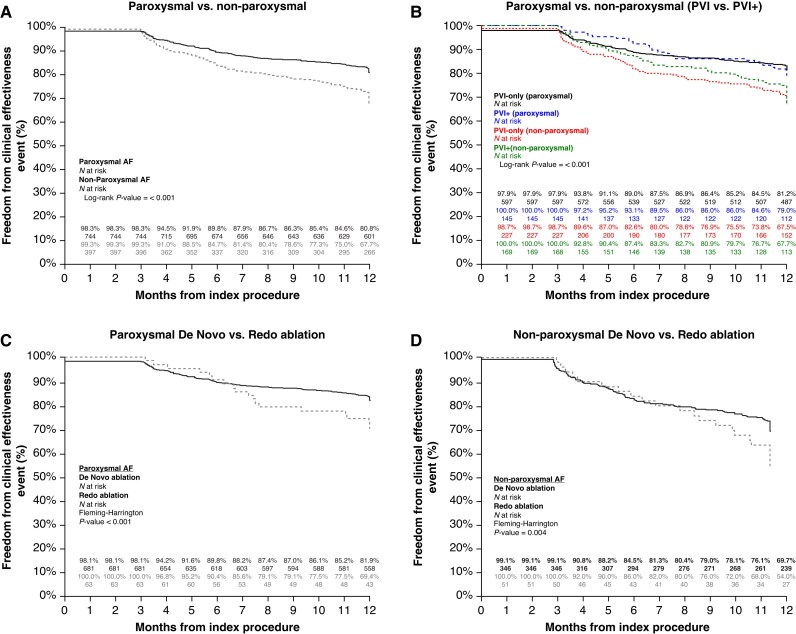
Twelve-month clinical effectiveness in (*A*) paroxysmal vs. non-paroxysmal AF patients and (*B*) further divided by lesion set (PVI vs. PVI+). (*C, D*) 12-month clinical effectiveness for *de novo* vs. redo ablation patients divided by AF indication.

When we look at clinical effectiveness, defined as freedom from arrhythmia recurrence, repeat procedure, and cardioversion, the overall 1-year effectiveness estimate was 76.3%. Differences in primary (off-AAD) and clinical (on-AAD) effectiveness are shown in Supplementary material online, Figure 1. Separating paroxysmal AF and non-paroxysmal AF patient populations, clinical effectiveness was 80.8% and 67.7%, respectively (*Figure 3A*; *P* < 0.01). When further stratified by ablation strategies beyond PVI (PVI+), no benefit was seen in the clinical outcomes within AF indication. For paroxysmal AF patients, PVI-only vs. PVI+ resulted in 81.2% and 79.0% Kaplan–Meier estimates, respectively. Non-paroxysmal AF patients had 67.5% clinical effectiveness following PVI-only vs. 67.7% for PVI+. Notably, for this stratification by ablation strategies, the PVI+ group includes all combinations of lesion sets delivered beyond the PVs.

### *De novo* vs. redo atrial fibrillation ablation

Most patients treated in the FARADISE study were undergoing their first ablation procedure (90.1% *de novo*). Clinical effectiveness outcomes in these *de novo* ablation patients were compared to those receiving a redo ablation (*Figure* *3C* and *D*). *De novo* ablations had significantly better outcomes compared to redo ablations in paroxysmal (81.9% and 69.7%, *P* < 0.01) and non-paroxysmal AF (69.4% and 54.0%, *P* < 0.01). When further evaluating the *de novo* ablation patient population, there was no significant difference in long-term outcomes based on the lesion set delivered. In the *de novo* paroxysmal AF patients, clinical effectiveness was 81.2% for PVI-only (*n* = 580) and 83.1% for PVI+ (*n* = 114). The *de novo* non-paroxysmal AF group was more evenly split between PVI-only (*n* = 222) and PVI+ (*n* = 127) with no difference in 1-year clinical effectiveness (67.7% and 72.2%, respectively).

### Predictors of 1-year success

**Figure 4:**
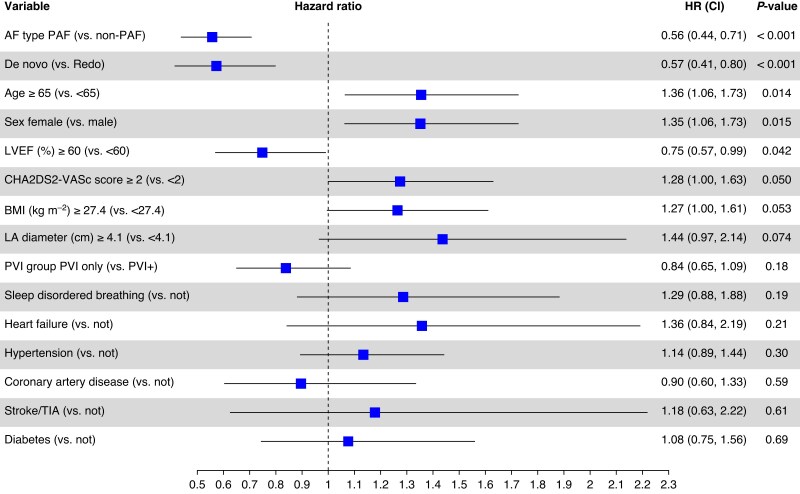
Forest plot identifying predictors of 1-year clinical effectiveness. Hazard ratios >1 indicate increased risk for treatment failure (arrhythmia recurrence, repeat ablation, or cardioversion).

Clinical characteristics were evaluated for their impact on 1-year clinical effectiveness, and results are shown in *Figure 4*. The two strongest, independent predictors of success following PFA were having paroxysmal AF (0.56 [0.44, 0.71], *P* < 0.01) and undergoing a *de novo* ablation procedure (0.57 [0.41, 0.80], *P* < 0.01). Additional factors associated with improved long-term outcomes includes age <65, male sex, and left ventricular ejection fraction of >60%. In this uni-variable analysis, both BMI (<27.4 kg/m^2^; *P* = 0.050) and CHADS-VASc (<2; *P* = 0.053), trended towards better outcomes, but did not reach significance. Aligned with the Kaplan–Meier results shown in *Figure 3B*, the lesion set (PVI-only vs. PVI+) was not a significant predictor of 1-year outcomes (*P* = 0.18). The final Cox proportional hazards multi-variable model included three components: paroxysmal AF indication vs. non-paroxysmal AF (0.44 [0.29, 0.66], *P* < 0.01), *de novo* vs. redo (0.50 [0.30, 0.82], *P* = 0.007), and BMI ≥27.4 kg/m^2^ (1.62 [1.06, 2.47], *P* = 0.026).

### Learning curve

**Figure 5:**
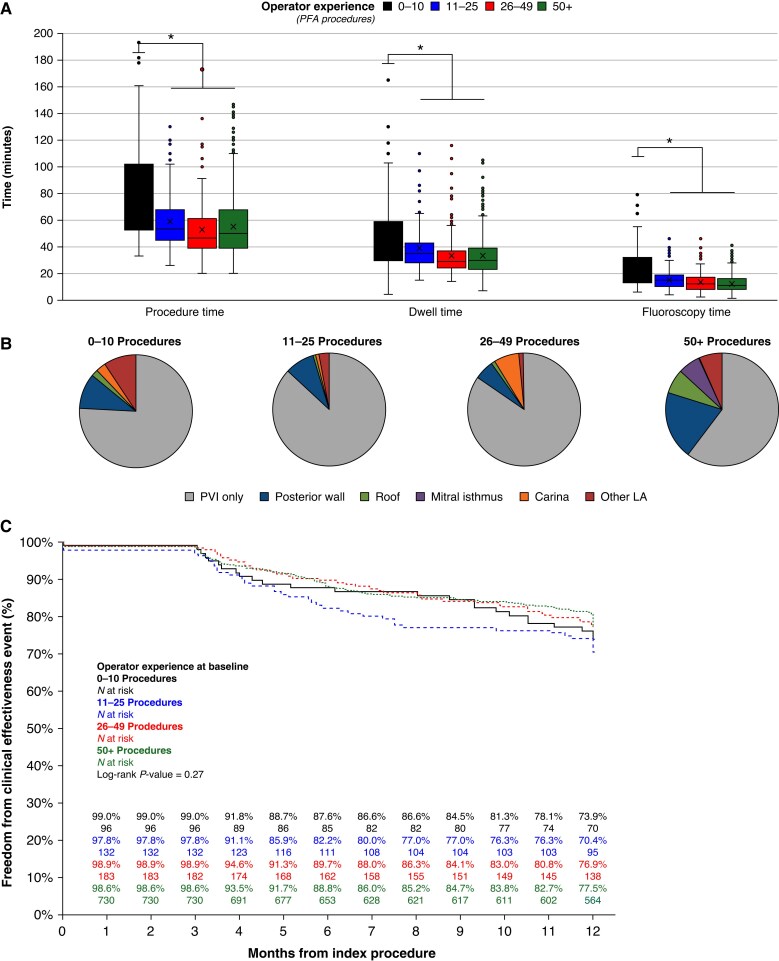
Learning curve evaluation. (*A*) Procedure times, (*B*) ablation set, and (*C*) 12-month clinical effectiveness by operator experience. * Indicates *P* < 0.01.

To evaluate learning curve, the 134 operators participating in this registry were grouped in four strata based on number of prior pentaspline PFA procedures performed: ≤ 10; 11–25; 26–49; 50+ procedures. Operators who had performed more than 50 PFA procedures prior to the start of the FARADISE registry more often performed ablations beyond the PVs (*Figure 5*). Procedure, LA dwell, and fluoroscopy time significantly decreased as operator experience increased (*Figure 5*). Median skin-to-skin procedure time decreased from 75 [53, 102] minutes for operators who performed <10 prior PFA procedures, to 53 [45, 68], 47 [39, 61], and 50 [39, 68] for operators with 11–24, 26–49, and 50+ prior PFA cases, respectively. In the most experienced group (50+ procedures), there was a numerical increase in procedure and LA dwell times compared to the prior group (26–49 procedures), in part due to the additional ablations performed. However, this did not translate to increased fluoroscopy time (*Figure 5*). At 1-year, clinical effectiveness for freedom of arrhythmias did not differ by operator’s prior experience with the pentaspline PFA catheter. Across experience levels, 1-year success rates were 73.9%, 70.4%, 76.9%, and 77.5% for operators with ≤10, 11–25, 26–49, and 50+ procedures, respectively (*P* = 0.27).

## Discussion

The FARADISE registry provides prospective evaluation of real-world use of the pentaspline PFA system in performing AF ablation, with insights on procedural practice and outcomes. Long-term follow-up is ongoing, but the 1-year results demonstrate favourable procedural and safety outcomes across geographies and operators in one of the largest prospective PFA registries to date. Clinical effectiveness at 1-year is promising, with a higher overall effectiveness in the paroxysmal AF group. The ablation strategy does not appear to impact 1-year, single procedure effectiveness, although most patients in this registry were treated with a PVI-only approach. This registry is uniquely positioned to capture early experience with the commercial availability of the pentaspline PFA catheter and demonstrates efficiency improvements with experience. The short learning curve is evident from the decrease in procedure time based on operator experience, although this trend is offset at higher levels of experience where the use of additional PFA applications beyond the PVs increased. At 1-year, no significant differences are observed in the clinical effectiveness based on experience or ablation strategy, reinforcing the benefit of the short learning curve of the pentaspline PFA catheter.

### Procedural characteristics

Use of the pentaspline PFA catheter was relatively consistent across 21 countries. Most patients treated had paroxysmal AF, and the dominant ablation strategy was a PVI-only approach. However, with sicker, more complex patients, additional non-PV ablations were typically performed more often (19% in paroxysmal vs. 43% in non-paroxysmal AF; *Table 2*). This resulted in longer procedure times but did not improve clinical effectiveness at 12-months. These practice patterns are consistent with other studies characterizing use of the pentaspline PFA catheter.^7,9–11^ Expansion to more geographies, including the US, may demonstrate differing procedural workflows and preferred ablation targets. Moreover, the optimal dosing and application number for PFA for each ablation target to achieve a durable transmural lesion has not yet been established.

Another important consideration for PFA procedures is the sedation strategy. Currently, the guidelines do not prescribe a specific strategy for AF ablation procedures,^2^ while sedation options are often dictated by geographical regulations and institutional requirements and experience. Recent trends have shown general anaesthesia to be the most used technique, though use of deep sedation is increasing.^12^ This is consistent with the experience captured in FARADISE. The most common approach was general anaesthesia; however, operators from nine countries (43%) used deep sedation for at least some of their PFA cases, with two countries (10%) solely using deep sedation. Several recent studies have demonstrated effective use of deep sedation protocols with pentaspline PFA.^13–16^ However, the optimal strategy is still unknown and warrants further investigation. With the introduction and evolution of PFA technologies in the AF ablation space, we may see changes in standard practice and sedation strategies not previously possible or preferred with the use of thermal ablation technologies.

### Safety

Overall, this study has demonstrated a very favourable procedural safety profile with an early onset device- and procedure-related SAE rate of 1.5%. Further, this finding was not dependent on the lesion set delivered, i.e. additional lesions performed outside of the PVs did not increase the rate of SAEs. At 12-month follow-up, there were no reported instances of atrio-oesophageal fistula, PV stenosis or oesophageal lesions, with only a single partial phrenic nerve paresis. These findings are consistent with other large clinical trials and PFA registries.^9,11,17,18^ It appears that pentaspline PFA can be safely used by operators with any level of experience, without the continuous need for oesophageal temperature measurements, phrenic nerve pacing capture, or visual monitoring of catheter position in the PV antrum that is recommended for thermal ablation modalities.

#### Cerebrovascular events

In the SAEs reported from the 1158 AF patients, there was one instance of stroke (0.1%) and one instance of TIA (0.1%) following the PFA procedure. This low rate of cerebral complications in this study is consistent with other registries^9,11^ as well as more rigorous evaluations performed in clinical trials.^17,19,20^ The ADVENT trial reported a single stroke event in the thermal arm and no symptomatic neurological events in the PFA arm,^17^ and a low rate of silent cerebral events following PFA in a paroxysmal AF patient population.^19^ Similarly, in non-paroxysmal AF patients, there was only one patient with a post-procedural silent cerebral embolism and no instances of stroke or TIA.^20^ Performing a cardiovascular procedure in the systemic circulation unavoidably carries a baseline risk of cerebral or systemic embolization, regardless of the type of procedure.

#### Haemolysis

There were four instances of haemolysis reported in this registry (one paroxysmal and three non-paroxysmal AF patients) after 66, 93, 140, and 161 PFA applications. It is important to note that clinical outcomes via standard-of-care monitoring were evaluated, and the rate of haemolysis based on positive biomarkers was not studied prospectively. However, what we observe here is consistent with prior observations where those at risk for haemolysis were non-paroxysmal AF patients with a higher number of PFA applications delivered during the procedure.^21,22^ Venier *et al*. found that ∼70 PFA applications was predictive of haemolysis, with Popa *et al*. suggesting 54 PFA applications or more reach a significant level for hemolysis.^22,23^ There is limited evidence as to the long-term sequalae for these acute events; however, this does force the operator to consider the balance between ablating sufficient tissue to treat the arrhythmia without over-ablating. So far, randomized controlled trials testing ablation targets beyond the PVs show only humble benefits, although a recent single-arm study demonstrated 73% effectiveness at 1-year.^24^ Aside from the number of applications, it is also important to consider contact, as improved tissue contact results in a larger amount of the electric field applied to the myocardium and reduces the exposure to the blood pool.^21^ Adjunctive imaging modalities (3D mapping, ICE) or contact assessment technologies may help limit haemolysis seen after PFA procedures. Physicians may also consider peri-procedural or immediate post-procedural fluid infusion to help prevent haemolysis in selected patients, which could be based on patient characteristics or anticipated lesion set.^21,25^ Further evaluation is needed to understand the incidence of haemolysis and the risk factors associated with PFA.

#### Coronary spasm

Several reports have published on the occurrence of coronary spasms following PFA, both proximity-related and, in rare instances, generalized.^9,26,27^ In this study, only 7.2% and 4.4% of cases performed a CTI or MI ablation, respectively, with a majority of them using the pentaspline PFA catheter to perform the linear ablations. Despite the use of PFA to perform these linear ablations, there was only one reported instance of proximity-related coronary spasm in a patient receiving PVI + CTI ablation with no sequelae. While there was no systematic coronary evaluation performed after PFA ablation in the vicinity of the coronary arteries, there were no reports of the clinical need for such investigations based on symptoms. However, this does not exclude any (sub-clinical) minor spasms as there was no additional coronary angiography performed during ablation.

### One-year clinical efficacy

FARADISE provides one of the largest, *prospective* real-world outcomes of AF ablation using the pentaspline PFA catheter and, importantly, includes over 40 centres and over 130 operators. The 1-year clinical effectiveness demonstrated in the FARADISE registry is similar to other clinical trials and other real-world experiences with pentaspline PFA. The strict endpoints, incorporating medication use as treatment failure and the 30-second arrhythmia recurrence definition, account for most of the composite endpoints met in this study. Clinical effectiveness provides a more representative outcome of treatment success based on documented recurrence or the need for clinical intervention (cardioversion or repeat ablation). In this study, clinical effectiveness reached 81% for paroxysmal AF and 68% for non-paroxysmal AF patients, which is aligned with previous reports from registries^8,9^ and clinical trials.^24,28^ In the ADVENT RCT, 73% and 71% of patients in the PFA and thermal groups were free from recurrence (off AADs) at 1 year. Large thermal ablation registries have also reported higher on-AAD, 1-year effectiveness in paroxysmal vs. persistent AF patients.^17^ The ESC-EHRA AF ablation long-term registry reported 75% and 71% of paroxysmal and persistent patients free of arrhythmia recurrence at 1 year, respectively.^29^ Similarly, the Cryo AF Global registry reported success rates of 86% in paroxysmal AF and 71% in persistent AF.^30^ Long-term follow-up is still ongoing with patients followed via institutional standard-of-care out to 3 years.

There were no significant differences in outcomes within indication based on the lesion set delivered. However, procedures were performed based on operator’s standard-of-care, and lesion sets were decided at the discretion of the operators. PVI-only was the predominate ablation approach used regardless of AF indication, and comparisons to PVI+ shown here are limited. Other registries have looked at outcomes based on lesion sets in non-paroxysmal AF patients with no significant difference in clinical outcomes.^31,32^ Several randomized studies are ongoing to evaluate outcomes after PFA based on lesion sets.

### Learning curve

Operators in FARADISE had a wide variety of experience with the pentaspline PFA catheter. The short learning curve evident here was similar to that demonstrated in clinical trials and real-world evidence.^7,9,10,33^ FARADISE showed improvements in procedural efficiency after the first ten PFA procedures. However, that effect decreased slightly in our most experienced group (50+ PFA procedures) in part due to the increase in non-PV ablations performed by these operators. These operators are likely to be performing AF ablation on the more complex, sicker patient populations which are perceived to need additional lesions or have challenging anatomies leading to longer procedure times. One-year outcomes reported here found no significant difference in clinical effectiveness of a single PFA procedure based on operator experience with the pentaspline PFA catheter prior to enrolling patients in FARADISE. These findings are aligned with the experience captured by the early adopters of the PFA technology in the EU-PORIA registry^9^ and reinforce the idea of seamless integration of pentaspline PFA procedures into clinical practice. While the current data suggest a minimal effect of learning curve on procedural outcomes with pentaspline PFA, most procedures were performed in centres with well-trained electrophysiologists proficient in AF ablation with other technologies. There is still a need for consistent and rigorous training for operators new to ablation for AF, and specifically with PFA, considering the various available systems and form factors to ensure optimal outcomes and avoid underestimation of risks, over-ablating cardiac structures, and potential related complications.^34^

### Future direction

These findings reinforce the early experience with the pentaspline PFA catheter as a result of unprecedented adoption of PFA in the electrophysiology lab. The efficiency and favourable safety profile have made PFA a promising alternative for cardiac ablation.^35^ With each PFA system and catheter form factor, further research is needed to understand optimal workflows, dosing, and durability similar to work previously performed with thermal ablation technologies.^36^ Future development of mapping solutions or acute measures of lesion durability will aid in performing more precise PFA procedures and may help eliminate gaps.^37^ For example, although not available at the time of this registry, the second generation pentaspline PFA catheter is navigation-enabled and mapping-integrated (FARAVIEW™, Boston Scientific Inc.). Further study is warranted to understand its potential to reduce procedure and fluoroscopy times, improve catheter guidance and tissue contact, and shorten the learning curve.

## Limitations

There are several limitations inherent in this real-world, standard-of-care registry. This is a single-arm study with no comparative ablation modality. Procedures, lesion numbers and dosing, and strategies beyond PVI were performed per hospital’s standard-of-care and physician’s discretion. Randomized studies such as PIFPAF-PFA (NCT04524364) and DOPPIO (NCT07021313) are underway to evaluate ablation strategies and dosing. However, this does provide insight on everyday use of the device in clinical settings. Notably, there is low use of 3D mapping and ICE in the procedures (primarily PVI-only) captured in this registry, which may not reflect use in more complex ablation cases. Given the recent development of dedicated mapping options with the OPAL HDx system, future research will show how this may optimize workflow and outcomes. Further, there is significant heterogeneity across operator experience which could impact acute procedural outcomes. Lastly, this report details only the findings up to 12 months from this registry. Follow-up is ongoing, with patients followed out to 3 years to evaluate long-term efficacy. This data are expected to be available in 2027.

## Conclusion

This prospective global registry captures the evolution in procedural use and outcomes of the pentaspline PFA catheter in routine clinical care beyond the early adoption of the technology. Real-world use of the pentaspline PFA catheter demonstrated favourable acute procedural and safety outcomes and 1-year clinical effectiveness across geographies and operators. PVI-only was the predominant ablation strategy regardless of AF indication, while no differences were observed at 1-year between ablation strategies within indications. Increased operator experience in this registry led to more efficient procedure times despite more complex cases with lesions more often delivered beyond PVI.

## Supplementary Material

euaf182_Supplementary_Data

## Data Availability

The data and study protocol for this clinical study may be made available to other researchers in accordance with Boston Scientific’s data sharing policy (http://www.bostonscientific.com/en-US/datasharing-requests.html).
