# Supplementary material for: Real-world experience with the pentaspline pulsed field ablation system: one-year outcomes of the FARADISE registry
Source: Europace. 2025 Sep 1;27(9):euaf182. doi: 10.1093/europace/euaf182 (PMC12400809; doi:10.1093/europace/euaf182)
Supplement: euaf182_Supplementary_Data [file euaf182_supplementary_data.docx]

**Real-World experience with the Pentaspline Pulsed Field Ablation System:**

**One-year outcomes of the FARADISE Registry**

Lucas V.A. Boersma^1^, Gábor Széplaki^2^, Antonio Dello Russo^3^, Ignacio García-Bolao^4^, Michael Efremidis^5^, Nándor Szegedi^6^, Stephan Willems^7^, Haris Haqqani^8^, Estelle Gandjbakhch^9^, Francesco Solimene^10^, George Andrikopoulos^11^, Martin Fiala^12^, Pascal Defaye^13^, Armin Luik^14^, Patrick Lugenbiel^15^, Lars Eckardt^16^, Alexandre Ouss^17^, Jean-Manuel Herzet^18^, Javier Ramos Maqueda^19^, Sélim Abbey^20^, Joaquín Osca^21^, Azlan Hussin^22^, Nele Cielen^23^, Madeline Johnson^23^, Elizabeth M. Albrecht^23^, Brad S. Sutton^23^, Johan Vijgen^24^

**Table S1. FARADISE Registry – Clinical Investigators and Sites**

| **Principal Investigator** | **Site** |
| --- | --- |
| Sélim Abbey | Hôpital Privé du Confluent SAS, Nantes, France |
| Pedro Adragão | Hospital de Santa Cruz, Lisbon, Portugal |
| George Andrikopoulos | Henry Dunant Hospital Center, Athens, Greece |
| Paweł Balsam | Uniwersyteckie Centrum Kliniczne Warszawskiego Uniwersytetu Medycznego, Warsaw, Poland |
| Leonard Bergau | Georg-August-Universitaet Göttingen, Göttingen, Germany |
| Stefano Bianchi | Ospedale Isola Tiberina Gemelli Isola, Rome, Italy |
| Lucas VA Boersma (Global PI) | St. Antonius Ziekenhuis, Nieuwegein, Netherlands |
| Fayez Bokhari | King Fahd Armed Forces Hospital, Jeddah, Saudi Arabia |
| Serge Boveda | Clinique Pasteur, Toulouse, France |
| Alan Bulava | Nemocnice České Budějovice a.s., České Budějovice, Czech Republic |
| Joseph Yat Sun Chan | Prince of Wales Hospital, Hong Kong, China |
| Pascal Defaye | CHU Grenoble - Hôpital Michallon, Grenoble, France |
| Antonio Dello Russo | AOU delle Marche - PO GM Lancisi, Ancona, Italy |
| Mattias Duytschaever | St. Jan Hospital, Bruges, Belgium |
| Lars Eckardt | University Hospital of Muenster, Muenster, Germany |
| Michael Efremidis | Onassis Cardiac Surgery Center, Athens, Greece |
| Martin Fiala | Neuron Medical in Brno, Brno, Czech Republic |
| Estelle Gandjbakhch | Sorbonne Université, APHP, Pitié-Salpêtrière Hospital, Institute of Cardiology, ICAN Institute for Cardiometabolism and Nutrition, Paris, France |
| Ignacio García-Bolao | Clinica Universidad de Navarra, Pamplona, Spain |
| Michael Gramlich | Universitaetsklinikum Aachen (UKA), Aachen, Germany |
| Dhiraj Gupta | Liverpool Heart and Chest Hospital, Liverpool, United Kingdom |
| Yves Guyomar | Hôpital Saint Philibert, Lomme, France |
| Haris Haqqani | The Prince Charles Hospital, Brisbane, Australia |
| Jean-Manuel Herzet | CHR La Citadelle, Liège, Belgium |
| Azlan Hussin | Institut Jantung Negara, Kuala Lumpur, Malaysia |
| Nicholas Jackson | John Hunter Hospital, Newcastle, Australia |
| Decebal-Gabriel Lațcu | Centre Hospitalier Princesse Grace, Monaco, Monaco |
| Antoine Lepillier | Centre Cardiologique du Nord, Saint-Denis, France |
| Patrick Lugenbiel | Universitaetsklinikum Heidelberg, Heidelberg, Germany |
| Armin Luik | Staedtisches Klinikum Karlsruhe, Karlsruhe, Germany |
| Ruggero Maggio | Ospedale di Rivoli, Rivoli, Italy |
| Heikki Makynen | Tays Sydansairaala Tampere, Tampere, Finland |
| Claire Martin | Papworth Hospital, Cambridge, United Kingdom |
| Javier Moreno | Hospital Universitario Ramón y Cajal, Madrid, Spain |
| Joaquín Osca | Hospital Universitario La Fe, Valencia, Spain |
| Alexandre Ouss | Catharina Ziekenhuis, Eindhoven, Netherlands |
| Vincent Edward Paul | Fiona Stanley Hospital, Murdoch, Australia |
| Javier Ramos Maqueda | Hospital Clinico Universitario Lozano Blesa, Zaragoza, Spain |
| Antonio Rossillo | Ospedale San Bortolo di Vicenza Azienda ULSS 8 Berica, Vicenza, Italy |
| Raphael Rosso | Tel Aviv Sourasky Medical Center, Tel Aviv, Israel |
| Francesco Solimene | Casa Di Cura 'Montevergine' S.P.A., Mercogliano, Italy |
| Stefan Spitzer | Facharztzentrum Dresden-Neustadt Betriebsgesellschaft mbH - Zentrum fuer klinische Pruefungen, Dresden, Germany |
| Stefan Stojkovic | Allgemeines Krankenhaus AKH, Vienna, Austria |
| Nándor Szegedi | Semmelweis University, Cardiovascular Center, Budapest, Hungary |
| Gábor Széplaki | Mater Private Hospital, Dublin, Ireland |
| Olivier Thomas | Clinique Ambroise Paré, Neuilly-sur-Seine, France |
| Hung Fat Tse | Queen Mary Hospital, Hong Kong, China |
| Johan Vijgen | Hartcentrum Hasselt Jessa Ziekenhuis Campus Virga Jesse, Hasselt, Belgium |
| Stephan Willems | Asklepios Klinik Saint Georg, Hamburg, Germany |

**Table S2. Additional clinical details for the hemolysis cases in the FARADISE registry.**

|  | **Case #1** | **Case #2** | **Case #3** | **Case #4** |
| --- | --- | --- | --- | --- |
| **AF Indication** | Persistent | Persistent | Persistent | Paroxysmal |
| **Number of PFA applications** | 93 | 66 | 161 | 140 |
| **Lesion Set** | PVI + MI | PVI | PVI + PW + MI | PVI |
| **ICE Use** | No | No | No | No |
| **Clinical Symptoms** | Hemoglobinuria | Hematuria | Hematuria | Urinary Retention |
| **Lab Results** | Decreased Haptoglobin 0.07g/L  Increased LDH 667 IU/L | Decreased Haptoglobin 0.02 g/L  Increased bilirubin 41 umol/L | Decreased Haptoglobin 0.03 g/L  Increased bilirubin 41 umol/L | *Total bilirubin peaked 110 umol/L  Direct bilirubin 26 umol/L  LDH 572 U/L, AST peaked 260 u/l,  ALT peaked 107 u/l,  free hemoglobin 0.08 g/L |
| **Was Dialysis Required?** | No | No | No | No |
| **Hydration Protocol / Treatment** | N/A | N/A | IV Fluids (Treatment) Per source 2L IV plus PO hydration | N/A |
| **Additional Case Notes** |  |  | Acute kidney injury. Creatinine 94umol/L pre procedure with a peak 344umol/L noted post. Resolved at discharge. | Site reported hemolysis likely due to Rocephin |

*lab sample was hemolyzed which may affect results

**Figure S1. Kaplan-Meier estimates for primary (off-AAD) and clinical (on-AAD) effectiveness for (A) all AF patients and (B) stratified by AF inidcaiton.**

**A)**

**B)**
